# A Point Prevalence Survey of Antimicrobial Use at Geita Regional Referral Hospital in North-Western Tanzania

**DOI:** 10.3390/pharmacy11050159

**Published:** 2023-10-07

**Authors:** Lutugera Kihwili, Vitus Silago, Emiliana N. Francis, Vicent A. Idahya, Zabron C. Saguda, Siana Mapunjo, Martha F. Mushi, Stephen E. Mshana

**Affiliations:** 1 Department of Pharmaceutical Sciences, School of Pharmacy, Catholic University of Health and Allied Sciences, Mwanza P.O. Box 1464, Tanzania; lutugerak@gmail.com; 2 Department of Microbiology and Immunology, Weill Bugando School of Medicine, Catholic University of Health and Allied Sciences, Mwanza P.O. Box 1464, Tanzania; mushimartha@gmail.com (M.F.M.); stephen72mshana@gmail.com (S.E.M.); 3 Ministry of Health, Dodoma P.O. Box 573, Tanzania; emmyfra@yahoo.com; 4 Geita Regional Referral Hospital, Geita P.O. Box 40, Tanzania; idahyav@yahoo.com (V.A.I.); czabrondr@gmail.com (Z.C.S.); 5 National Multi-Sectoral Coordinating Committee, Ministry of Health, Dodoma P.O. Box 573, Tanzania; smapunjo@yahoo.com

**Keywords:** antibiotic use, antimicrobial resistance, point prevalence survey, WHO-AWaRe classifications, WHO point prevalence survey

## Abstract

We conducted a point prevalence survey (PPS) to determine the prevalence of antibiotic use at Geita Regional Referral Hospital (GRRH) located along the shores of Lake Victoria in north-western Tanzania. This has led to the identification of gaps for improvement. This PPS study was conducted on 9–10 March 2023. Patient-related information, including sociodemographic and clinical data, was collected from medical records. STATA software version 15.0 was used to perform descriptive data analysis. About 94.8% (55/58) patients were on antibiotics with a mean (±SD) prescription of 2 (±0.5) antibiotic agents ranging from 1 to 4 different agents. The commonest indications of the antibiotic prescription were medical prophylaxis 47.3% (26/55) followed by empiric treatment 41.8% (23/55). In total, 110 prescriptions were made, of which metronidazole (25.5%; *n* = 28), ceftriaxone (23.6%; *n* = 26), and ampicillin–cloxacillin (23.6%; *n* = 26) were frequently observed. Only 67.3% (*n* = 74) of prescriptions complied with Tanzania Standard Treatment Guidelines. Moreover, according to the WHO-AWaRe classification, 50.9%, 23.6%, and 25.5% were under the Access category, Watch category, and Not Recommended category, respectively. The prevalence of antibiotic use among patients admitted to GRRH was high, whereby medical prophylaxis and empiric treatment were the commonest indications for antibiotic prescription. To support rational therapy and antimicrobial stewardship initiatives, we recommend that laboratories in regional hospitals be equipped to conduct sustained routine culture and antimicrobial susceptibility testing.

## 1. Introduction

Antimicrobial resistance (AMR) has been declared one of the top 10 global public health threats [1]. Microbes such as bacteria, viruses, fungi, and parasites undergo spontaneous genomic change over time or acquire mobile genetic elements that confer their resistance towards antimicrobials [2]. Consequently, AMR has made infections harder to treat, increasing the risk of spreading resistant bacterial strains and the severity of infections as well as mortality [1].

To combat the crisis of AMR, in May 2015, the World Health Assembly adopted a global action plan on antimicrobial resistance (GAP-AMR), which was officially published in January 2016 [3]. Optimizing the use of antimicrobial medicines in human and animal health was the fourth among the five major objectives of GAP-AMR [3]. Furthermore, the Tanzania Ministry of Health adopted and launched its 5 years rolling National Action Plan on Antimicrobial Resistance 2017–2022 (NAP-AMR 2017–2022) [4]. The 2017–2022 NAP-AMR has been revised to the 2023–2028 NAP-AMR to ensure the continuation of AMR activities [5]. Both NAP-AMRs list the optimization of antimicrobial use in human, animal, and plant health as the main strategic objective [4,5].

AMR is a complex crisis that is a result of several factors, including overprescribing and dispensing of antimicrobials, poor infection prevention and control, and lack of surveillance systems and diagnostic tests in healthcare facilities [6]. Misuse and overuse of antimicrobials are considered the main drivers in the development of AMR [1,7]. Infectious diseases often require treatment with antimicrobials; however, the burden of antimicrobial use is higher in low- and middle-income countries (LMICs) [8,9]. This consequently creates antimicrobial selection pressure on pathogens, resulting in the emergence of antimicrobial-resistant pathogens that may spread across health patients, health facilities, and communities due to poor infection prevention and control (IPC) [6,8,9]. Therefore, healthcare facilities provide excellent settings for the collection of hospital data to understand antimicrobial prescribing practices and antimicrobial use [6]. This may lead to the identification of targets for improvement with subsequent implementation of interventions aimed at reducing AMR through optimization of antimicrobial use [6].

In 2018, WHO developed a global methodology, namely the WHO Point Prevalence Survey (WHO-PPS), for the collection of data on antimicrobial use and monitoring while providing information on antimicrobial consumption at national and global levels [6]. This methodology encourages the standardization of antimicrobial use and facilitates the comparison of antimicrobial use between hospitals across districts, countries, and regions over time [6]. The WHO-PPS methodology collects sociodemographic and associated clinical information of all hospitalized patients from medical records relevant to patients’ treatment and management of infectious diseases even though, at the time of data collection, these patients are on antibiotic treatment [6]. Moreover, the WHO-PPS has a well-established methodology that aims to establish antimicrobial stewardship (AMS) and identify areas of improvement in healthcare facilities [10,11].

In Tanzania, PPS studies conducted in 2016 and 2019, before the outbreak of COVID-19, have reported an overall prevalence of antibiotic use among patients admitted to different hospitals ranging from 44.0% to 62.3% [11,12]. Ceftriaxone, metronidazole, and antibiotic agents in the penicillin class have been reported as the most frequently prescribed antibiotics in clinical practices [11,12]. The likelihood of antibiotic prescription and use was documented to be associated with patients being children (<2 years) and admission in surgical and pediatric wards [12]. During the outbreak of COVID-19, the irrational use of antimicrobials for treating symptoms related to COVID-19 increased; this significantly increased the antibiotic prescribing practices and prevalence of antibiotic use in hospital settings [13]. For instance, a systematic review and meta-analysis study by Malik and Mundra conducted between December 2019 and December 2021 documented that 78% of COVID-19 patients have been prescribed an antibiotic without proper clinical rationale [14]. Ceftriaxone (30.1%) and azithromycin (26%) were the commonest prescribed antibiotics among COVID-19 patients [14]. However, the latter was contrary to a study by Wang et al. that was conducted during the COVID-19 outbreak between January 2019 and December 2021 in China and observed a decreased antibiotic use among patients by 32.04% in 2020 and by 16.69% in 2021 compared to 2019, respectively [15]. 

Currently, we lack routine audits and reviews of available documents on antibiotic use among patients admitted to many regional hospitals in Tanzania, including Geita Regional Referral Hospital (GRRH). Consequently, this restrains our knowledge of monitoring and optimizing antimicrobial use and antimicrobial stewardship in regional referral hospitals. Additionally, the outbreak of the COVID-19 pandemic may have influenced antimicrobial prescribing practices among healthcare professionals as well as increased the prevalence of antibiotic use among patients admitted to regional referral hospitals as observed in the community setting [16]. Specifically, Ndaki and colleagues reported that 89.43% of the community drug dispensers recommended antibiotics to mystery clients with urinary tract infection (UTI)-like symptoms, and 58.93% were willing to sell partial courses [16]. Therefore, we designed this study to determine the prevalence of antibiotic use at GRRH to provide baseline data that can be used to optimize use through AMS and regular audits.

## 2. Materials and Methods

### 2.1. Study Design, Duration, and Setting

This point prevalence survey was conducted on 9–10 March 2023 at Geita Regional Referral Hospital (GRRH). GRRH is a government-owned regional referral hospital found within Geita Town Council and serves as the referral hospital for Geita Region, which is located along the shores of Lake Victoria in Tanzania. GRRH is a referral hospital for the five districts of the Geita Region, namely Geita, Chato, Bukombe, Mbogwe, and Nyang’hwale, with an estimated catchment population of 2,539,144 people. GRRH has a 230-bed capacity and annual hospital admission of about 8989 patients. Moreover, GRRH is currently providing the following services: outpatient, inpatient, surgical, obstetrics and gynecology, dental, and TB.

### 2.2. Study Population and Selection Criteria and Participant Sample Size Considerations

All patients admitted in respective wards during sampling days (9–10 March 2023) were eligible for enrollment in the study. We ensured that patients’ enrollment and data collection in the respective ward were completed on the same PPS day. We excluded all patients who were admitted after 8:00 a.m. in the same sampling. The considerations of participant sample size did not apply to the current study since the hospital (study setting) has less than 500 beds.

### 2.3. Data Collection

Data were collected electronically by mobile data gathering app Epicollect5 [17] using the WHO-PPS forms [6]. A team of 4 selected healthcare professionals (Doctor, Nurse, Pharmacist, and Laboratory technician) from GRRH were extensively trained on data collection tools on 8 March 2023 before actual data collection on 9–10 March 2023. Training of the team was conducted by the PI of the study in collaboration with a representative from the Ministry of Health. Patient-related information, including sociodemographic data such as age and sex, clinical data such as malaria and HIV status, and antibiotic use status, were collected from the medical records of patients enrolled in the current study.

### 2.4. Quality Control

The electronic data entries were quality-checked for errors and coherence with diagnostic codes, ensuring ward-level data matched patient forms and antibiotic forms before approval by the team leader (SEM). Thereafter, data were extracted in the form of Microsoft Excel and imported into STATA version 15.0 [18] for analysis.

### 2.5. Data Interpretation, Management and Analysis

Data were coded and then imported into STATA software version 13.0 for analysis. Percentages and fractions were used to present categorical data, while mean (±SD) and median [IQR] were used to present continuous data. To determine adherence towards standard guidelines, we compared all recorded prescriptions against Standard Treatment Guidelines and the National Essential Medicine List for Tanzania Mainland 6th edition of 2021 (STG-NEMLIT 6th Ed. 2021) [19] and WHO-AWaRe classifications [20].

## 3. Results

### 3.1. The Distribution of Patients Enrolled during the Point Prevalence Survey at Geita Regional Referral Hospital

A total of 86 patients were admitted during sampling days, of which 60 patients’ medical records were eligible for enrollment; however, only 58 patients’ records were included in the study. The majority of patients’ medical files reviewed for this study belonged to patients who were admitted to the surgical male ward (12/58), pediatric ward (10/58), and surgical female ward (8/58) (Table 1).

### 3.2. Sociodemographic and Clinical Characteristics of Patients Admitted during Point Prevalence Survey at Geita Regional Referral Hospital

The median [IQR] age in years of patients whose medical files were collected and reviewed for the current study was 25.5 [1.7–36] years. About 51.7% (30/58) of medical files reviewed belonged to the male sex. About 18.9% (11/58) of patients were diagnosed with malaria, 15.5% (9/58) of patients had surgery during current admission, 15.5% (9/58) of patients had urinary indwelling catheters, 1.7% (1/58) of patients had a central-vascular line, and 1.7% (1/58) of patients were HIV seropositive. Moreover, the majority of patients had peripheral vascular catheters 98.3% (57/58) (Table 2).

### 3.3. Prevalence of and Indications for Antibiotics Use among Patients Admitted to Geita Regional Referral Hospital

**Figure 1:**
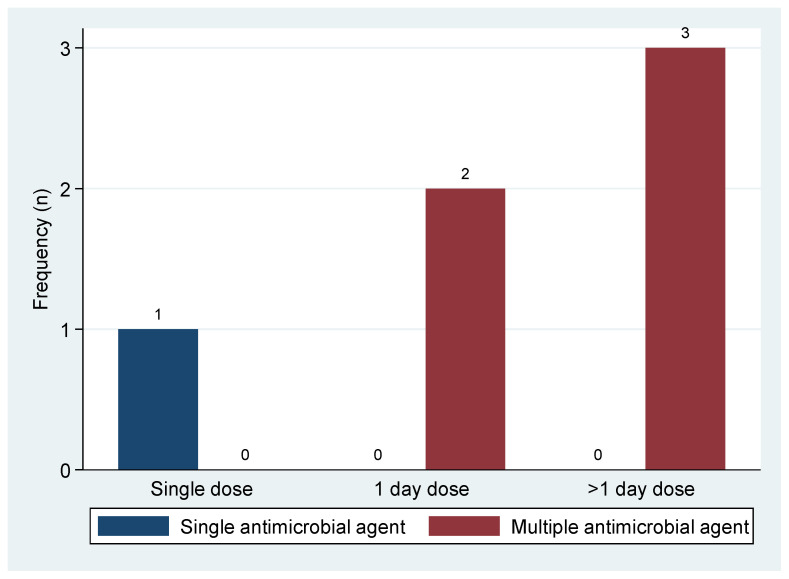
Dosage frequency and number of antibiotic agents for surgical prophylaxis among patients admitted to Geita Regional Referral Hospital. The blue bar in the histogram represents the number of patients who were administered one antibiotic agent once a day before surgery. Two red bars in the histogram represent the number of patients who were administered at least two antibiotic agents with multiple doses within a day and on more than one day, respectively.

About 94.8% (55/58) of patients were found to be using antibiotics during survey days, whereby the mean (±SD) number of antibiotic agents prescribed per patient was 2 (±0.5) with a range of one antibiotic agent to four different antibiotic agents. The commonest indication for the prescription of antibiotics was for medical prophylaxis 47.3% (26/55), followed by empiric treatment 41.8% (23/55). No sample was collected for culture and susceptibility testing. Two (8.7%) out of twenty-three patients on empiric treatment were on antibiotic prescriptions due to healthcare-associated infections (HCAIs), while the rest had community-acquired infections (CAIs) (Table 3). Moreover, six patients (10.9%) among those using antibiotics were on antibiotic exposure for surgical prophylaxis, of which three (50.0%) were on multiple antibiotic agents for more than one day (Figure 1). Furthermore, the overall number of antibiotic prescriptions on survey days was 110 prescriptions, of which the majority were parenterally administered (91.8%; *n* = 101). Metronidazole (25.5%; *n* = 28) followed by ceftriaxone (23.6%; *n* = 26) and ampicillin–cloxacillin (23.6%; *n* = 26) were frequently prescribed antibiotics (Table 3).

### 3.4. Compliance of Antibiotic Prescriptions with Standard Treatment Guidelines and National Essential Medicine List for Tanzania Mainland and WHO-AWaRe Classifications at Geita Regional Referral Hospital

**Figure 2:**
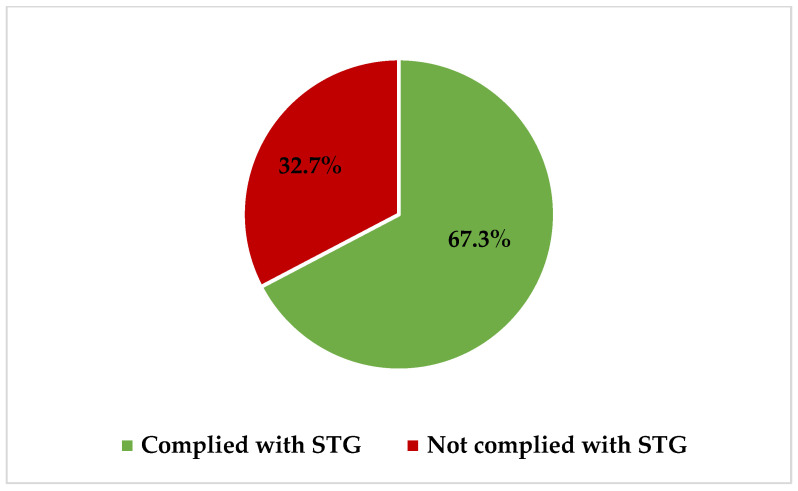
Percentage of compliance of antibiotic prescriptions with Standard Treatment Guidelines and National Essential Medicine List for Tanzania Mainland of 2021 (STG-NEMLIT 6th Ed. 2021) at Geita Regional Referral Hospital.

All antibiotics (100%; *n* = 110) prescribed at GRRH during PPS days were listed in STG-NEMLIT 6th Ed. 2021 for the treatment of various disease conditions. However, to be specific, only 67.3% (*n* = 74) of prescriptions complied with STG-NEMLIT 6th Ed. 2021, hence correctly prescribed for specific conditions (Figure 2).

**Figure 3:**
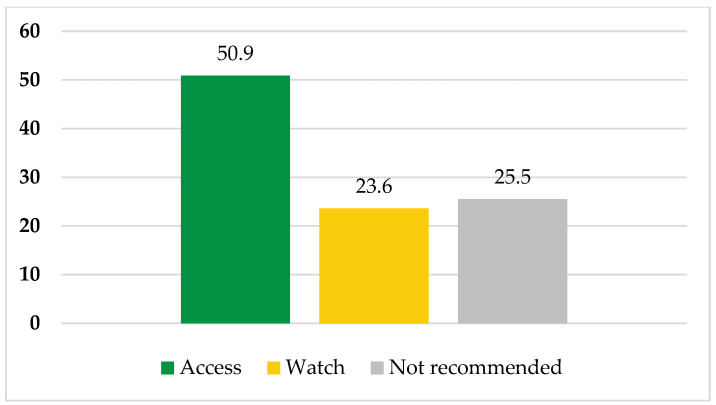
Percentage of compliance of antibiotic prescriptions with WHO-AWaRe classifications at Geita Regional Referral Hospital.

On the other hand, according to WHO-AWaRe classification, 50.9% (*n* = 56; amoxicillin, ampicillin, gentamicin and metronidazole) were under the WHO Access category, 23.6% (*n* = 26; ceftriaxone) were under the WHO Watch category, and 25.5% (*n* = 28; ampicillin–cloxacillin and ceftriaxone–sulbactam) were under the WHO Not Recommended category (Figure 3).

## 4. Discussion

To begin with, the prevalence of antibiotic use is high at GRRH. The majority of patients were on two or more antibiotics, and the commonest indications for antibiotic use were medical prophylaxis and empiric treatment. Culture and susceptibility testing was not performed. Moreover, about two-thirds and one-half of all prescriptions complied with standard treatment guidelines of Tanzania and WHO-AWaRe classification, respectively.

Moreover, on survey days 9–10 March 2023, a total of 86 patients were admitted to GRRH in antenatal and postnatal wards, neonatal wards, pediatric wards, and medical and surgical wards. Referral cases from lower healthcare facilities within the Geita Region accounted for 12.1%. Sixty patients were eligible for enrollment; however, only 58 patients’ medical records were reviewed for this study. The median [IQR] age of patients whose medical files were reviewed for the current study was 25.5 [1.7–36] years, ranging from 0 to 87 years. Moreover, the sex ratio of our study participants was almost one male to one female (51.7% vs. 48.3%). On the other hand, the proportions of patients with malaria diagnosis, surgical procedures, and urinary indwelling catheters ranged between 15% and 20%. Nearly all patients (98.3%) had indwelling peripheral vascular catheters as a quick and cost-effective method of vascular access among hospitalized patients [21,22] for the administration of medicines, fluids, and/or blood products [23,24].

In addition, contrary to previous studies in Tanzania and elsewhere [11,12,25,26], the prevalence of antibiotic use in the current study at GRRH was high (94.8%). For instance, a study by Horumpende et al. in 2020 reported an overall prevalence of antibiotic use of 44.0% among patients admitted to tertiary, regional, and district hospitals in the north-eastern region of Tanzania [11]. In line with our study setting (regional referral hospital), and to be specific, the prevalence of antibiotic use at regional referral hospitals in a study by Horumpende et al. was 59% [11]. Another similar study by Seni et al. in 2020 reported an overall prevalence of antibiotic use of 62.3% among patients admitted to two zonal referral hospitals and four regional referral hospitals [12], whereby the specific prevalence of antibiotic use at regional referral hospitals ranged from 65.7% to 74.3% [12]. Additionally, a study by D’Arcy and colleagues reported an overall prevalence of antibiotic use of 50.0% among patients admitted to hospitals in Ghana (55.0%), Uganda (45.0%), Zambia (57.0%), and Tanzania (30.0%) in 2021 [25]. Moreover, we observed that, on average, patients using antibiotics at GRRH were using at least two different agents, similar to a study by Seni et al., who documented that over half (54.8%) of patients using antibiotics were using two different agents [12]. Moreover, in the current study, patients with few antibiotic agents had one prescription, while those with more antibiotic agents had four prescriptions. The high use of antibiotics at GRRH in the current study increases the concern that GRRH may be a potential source of AMR. This observation warrants further studies on AMR infection and colonization among patients and contamination of inanimate hospital surfaces in this setting.

In the current study, the commonest indication for antibiotics prescriptions among patients admitted to GRRH was medical prophylaxis (47.3%), followed by empiric treatment (41.8%). The majority (91.3%) of patients on empiric treatment were on antibiotics for the treatment of community-acquired infections (CAIs). Additionally, Horumpende et al. [11] and Seni et al. [12] also previously observed and reported that treatment of CAIs was the most common reason for antibiotic use among those admitted to healthcare facilities in the same region. However, almost one-third of the patients in the studies by Horumpende et al. (30.0%) and Seni et al. (30.2%) were on antibiotics for surgical prophylaxis [11,12], unlike our study, wherein only 10.9% of patients on antibiotics at GRRH were prescribed antibiotics for surgical prophylaxis. The inclusion of zonal referral hospitals in the previous studies [11,12] may explain the high proportion of patients on surgical procedures and why they are most likely to be prescribed antibiotics for surgical prophylaxis. Furthermore, in the current study, the proportion of patients on empiric treatment was high (41.8%), and all had no sample collected for culture and sensitivity testing. It was also reported previously that only 2 of 591 patients on antibiotics were prescribed antibiotics based on culture and antimicrobial susceptibility testing results [12]. The main reason could be the lack of culture and antimicrobial susceptibility testing, despite the fact that in many healthcare facilities with culture and susceptibility services in the country, the utilization of services is still low. It should be noted that excessive empirical therapy promotes the emergence of resistant pathogens that can spread in health facilities and communities due to poor infection prevention control [27,28]. Therefore, this observation necessitates the establishment and implementation of culture and antimicrobial susceptibility testing in all district, regional, and zonal hospitals by the Ministry of Health in Tanzania.

Even though antibiotic use for surgical prophylaxis in the current study at GRRH is not as common compared to previous studies [11,12], almost 50% of patients using antibiotics for surgical prophylaxis were on multiple antibiotic agents, which was continued for more than one day. It was also reported previously that over 50% of patients using antibiotics for surgical prophylaxis received multiple antibiotic agents for more than three days [11]. This practice is, however, not in compliance with clinical practice guidelines for antimicrobial use for surgical prophylaxis, which recommend a single dose immediately before surgery, as documented previously by multiple studies [29,30,31]. From these observations, it is recommended that an antimicrobial stewardship team and program be established to implement the effective monitoring and optimization of antibiotic use at GRRH in compliance with local and national standard treatment guidelines. Further, this fulfills the priority action (antimicrobial stewardship) of strategic objectives number 5 of the National Action Plan on Antimicrobial Resistance 2023–2028 (NAP-AMR), which states that “Optimize the use of antimicrobial medicines in human and animal health” [5]. The coordinated antimicrobial stewardship program promotes rational antibiotic use, improves patient outcomes, and reduces the emergence and spreading of antimicrobial-resistant bacterial strains [32,33,34].

Furthermore, in the current study, the majority of prescribed antibiotics were administered parenterally (91.8%), in line with studies from Uganda (88.0%) and Kenya (63.4%), which observed that parenteral administration of antibiotics is a common practice among hospitalized patients [35,36]. Moreover, metronidazole, ceftriaxone, and antibiotic agents within the penicillin class, i.e., ampicillin, amoxicillin, and ampicillin–cloxacillin, were commonly prescribed as reported previously from studies in Tanzania [11,12], Uganda [35], and Kenya [36]. These commonly prescribed antibiotics are listed as first (e.g., ampicillin and amoxicillin) and second (e.g., ceftriaxone) treatment options in the standard treatment guidelines [19], thereby justifying their high use in this setting. We also observed that about one-third (32.7%) of all prescriptions in the current study at GRRH were not in compliance with STG-NEMLIT 6th Ed. 2021 [19]. This observation calls for an urgent establishment of antimicrobial stewardship teams that focus on the implementation of appropriate prescriptions of antibiotics as well as monitoring and optimizing antibiotic use in this setting. On top of that, one-half (50.9%) of all antibiotics, namely amoxicillin, ampicillin, gentamicin, and metronidazole, prescribed among patients admitted to GRRH belong to Access-classified antibiotics, whereas one quarter (i.e., ceftriaxone) belongs to Watch-classified antibiotics and another one-quarter (i.e., ampicillin–cloxacillin and ceftriaxone–sulbactam) belongs to Not Recommended-classified antibiotics by WHO-AWaRe classifications [20]. Our observations that Access-classified antibiotics are commonly prescribed are in line with previous studies from Tanzania, Uganda, and Kenya [12,35,36] and are supported by WHO recommendations [20].

The WHO recommends that Watch-classified antibiotics should be monitored and prioritized in antimicrobial stewardship programs [20]. The lack of culture and antimicrobial susceptibility testing at GRRH may explain the routine empirical prescriptions of Watch-classified antibiotics for treatments of different disease conditions. Moreover, the WHO does not recommend the use of a fixed-dose combination of multiple broad-spectrum antibiotics such as ampicillin–cloxacillin and ceftriaxone–sulbactam in clinical practice due to a lack of evidence-based studies. Therefore, non-compliance with STG-NEMLIT, routine prescription of Watch-classified antibiotics without culture and antimicrobial susceptibility testing, and prescription of Not Recommended-classified antibiotics at GRRH indicates inappropriate and irresponsible antimicrobial use in this setting. Consequently, it may be associated with an increased emergence and spreading of antimicrobial-resistant bacterial pathogens, as documented previously [27]. Therefore, as pointed out earlier, there is an urgent call to establish culture and antimicrobial susceptibility testing at all medical laboratories affiliated with district, regional, and zonal referral hospitals. Moreover, supportive supervision and mentorship of lower-tier laboratories by authorities, higher-tier laboratories, and/or microbiology experts are recommended to ensure the smooth and continuous running of culture and antimicrobial susceptibility testing. In turn, rational clinical management of patients can be enhanced, as well as improved patient outcomes and combatting the emergence and spreading of antibiotic-resistant bacterial pathogens.

## 5. Study Limitations

The current study is limited by the fact that a single PPS may be inadequate to provide information on prescription patterns and trends in antibiotic use at this facility.

## 6. Conclusions

The prevalence of antibiotic use among patients admitted to Geita Regional Referral Hospital was high (91.4%). The majority of patients were on two or more antibiotics, and medical prophylaxis and empiric treatment were the commonest indications for antibiotic use. Moreover, no sample was collected for culture and susceptibility testing. About two-thirds and one-half of all prescriptions complied with the standard treatment guidelines of Tanzania and WHO-AWaRe classification, respectively.

Therefore, medical laboratories at regional hospitals should be improved and supported to conduct sustainable routine culture and antimicrobial susceptibility testing for appropriate diagnosis of infectious diseases and guide rational management of patients. In addition, there is a need to establish an AMS team and program to enhance the appropriate prescription of antibiotics and implement effective monitoring and optimization of antibiotic use at regional referral hospitals to comply with local and national standard treatment guidelines. Moreover, further studies are warranted to evaluate the safety, effectiveness, and appropriate use of Not Recommended-classified antibiotics in routine clinical practices.

## Figures and Tables

**Table 1:** The distribution of patients enrolled during the point prevalence survey at Geita Regional Referral Hospital.

Ward	Survey Date	Total Patients	Eligible Patients	Enrolled Patients
Antenatal ward	9 March 2023	7	3	3
Pediatric 6	9 March 2023	10	10	10
Medical male	9 March 2023	5	4	4
Tuberculosis	9 March 2023	1	0	0
Medical female	9 March 2023	5	4	2
Postnatal ward	10 March 2023	7	5	5
Neonatal	10 March 2023	5	5	5
Pediatric 1	10 March 2023	10	5	5
Grade	10 March 2023	7	4	4
Surgical male	10 March 2023	16	12	12
Surgical female	10 March 2023	13	8	8
Total		86	60	58

**Table 2:** Sociodemographic and clinical characteristics of patients admitted during the point prevalence survey at Geita Regional Referral Hospital.

Characteristics	Category	Frequency (*n*)	Percentage (%)
Age in years	Median [IQR]	25.5 [1.7–36]	-
Sex	Female	28	48.3
	Male	30	51.7
Referral	No	51	87.9
	Yes	7	12.1
Central vascular line	No	57	98.3
	Yes	1	1.7
Peripheral vascular catheter	No	1	1.7
	Yes	57	98.3
Urinary indwelling catheter	No	49	84.5
	Yes	9	15.5
Intubation	No	58	100
	Yes	0	0.0
Malaria infection	No	21	36.2
	Yes	11	18.9
	Not diagnosed	26	44.9
Status of TB infection	No	11	18.9
	Unknown	47	81.1
HIV status	Negative	9	15.5
	Positive	1	1.7
	Unknown	48	82.8
Surgical procedure	No	49	84.5
	Yes	9	15.5

**Table 3:** Prevalence of and indications for antibiotic use among patients admitted to Geita Regional Referral Hospital.

Characteristic	Category	Frequency (*n*)	Percentage (%)
Currently using antibiotics	No	3	5.2
	Yes	55	94.8
Antibiotic agents	Mean (±SD)	2 (±0.5)	-
Reason/indication for antibiotic use (*N* = 55)	Medical prophylaxis	26	47.3
	Surgical prophylaxis	6	10.9
	Empirical treatment	23	41.8
Indications for empiric treatment (*N* = 23)	CAIs	21	91.3
	HCAIs	2	8.7
Number of prescriptions (*N* = 110)	Amoxicillin	2	1.8
	Ampicillin	6	5.5
	Ampicillin–cloxacillin	26	23.6
	Ceftriaxone	26	23.6
	Ceftriaxone–sulbactam	2	1.8
	Gentamicin	20	18.2
	Metronidazole	28	25.5
Route of administration of prescribed antibiotics (*N* = 110)	Oral	9	8.2
	Parenteral	101	91.8

## Data Availability

The data presented in this study are available on request from the corresponding author.
